# Supplementary material for: Patterns of Intron Gain and Loss in Fungi
Source: PLoS Biol. 2004 Nov 30;2(12):e422. doi: 10.1371/journal.pbio.0020422 (PMC532390; doi:10.1371/journal.pbio.0020422)
Supplement: Table S1 — Also available at http://genes.mit.edu/NielsenEtAl/. (4.3 MB ZIP). [file pbio.0020422.st001.zip › NielsenEtAl/html/152.html]

AN5915.1.NCU06447.1.MG06273.1.FG07288.1


```
 CLUSTAL W (1.82) Multiple Sequence Alignments - Introns Inserted


Sequence 1: MG06273.1	190 aa
Sequence 2: FG07288.1	190 aa
Sequence 3: NCU06447.1	190 aa
Sequence 4: AN5915.1	198 aa
Alignment Length: 201 aa
Number Identitical Residues: 114 aa
Alignment Score (without introns) 5755


MG06273.1 	MDSVELDQTPFAAVSAQTSRLSR0QYQAVLDQSTPYVLYRWIGTGITLVLFFARIFVAQG
NCU06447.1	METAEEPMTPFGTFTAQSNKLQR0QYQALLDQSTPYVTYRWVGTGVALFLFFVRVFVAQG
FG07288.1 	MNAPEPEQTPFEAVSVHTSRIQR0KYQALLDQSTPFVLYRWVGTVVCLVLFFLRILVAQG
AN5915.1  	MDVPEPEQTPFTAVSAQTSKLAR0QYQTYLDASTPFTAYRWIGTAVLLFIFFLRIILAQG
          	*:  *   *** :.:.::.:: * :**: ** ***:. ***:** : *.:** *:::***

MG06273.1 	WYI-~--------V1AYALGIYLLNLFLAFLQPKFDPSNEALDNDMEDGAVGG--LPTKQ
NCU06447.1	WYI-~--------V~AYALGIYLLNLFLAFLTPKFDPSSDALDTEMEDGSVGT--LPTKQ
FG07288.1 	WYI-~--------V1AYALGIYLLNLFLAFLQPKFDPSNEEADNDMEDGSVGT--LPTKS
AN5915.1  	WYIG1ITVTNGTTV~AYTVGIYLLNLFLLFLQPKFDPS-LTQDEGLEDGDAGAPSLPTKQ
          	***.  : :..::* **::********* ** ******    *  :*** .* .:****.

MG06273.1 	DEEFRPFIRRLPEFKFWHAATRAIVIGFLCSWFEVFNVPVFWPVLVMYWFILFFLTM1RK
NCU06447.1	DEEFRPFIRRLPEFKFWHSATRAVAISFLCSWFEIFNIPVFWPVLVMYWLMLFILTM1RK
FG07288.1 	DEEFKPFIRRLPEFKFWYWATRAIVIGFTCSWFEVFNVPVFWPVLVMYWFILFFLTM1RK
AN5915.1  	DDEFRPFIRRLPEFKFWHSATRALAIGFLCTWFSVFDIPVFWPVLVVYWILLFVLTM1RR
          	*:**:************: ****:.*.* *:**.:*::********:**::**.*** *:

MG06273.1 	QIQHMIKYRYVPFSFGKARYAKNSS
NCU06447.1	QIQHMIKYRYVPFTIGKARYNKNSN
FG07288.1 	QIQHMIKYRYVPFTVGKKNYAKNSS
AN5915.1  	QIQHMIKYRYVPFSFGKARYGRS--
          	*************:.** .* :.
```
